# A preliminary investigation of re‐evaluating the irradiation dose in hepatocellular carcinoma radiotherapy applying 4D CT and deformable registration

**DOI:** 10.1002/acm2.13111

**Published:** 2021-01-15

**Authors:** Hua Xu, Guanzhong Gong, Yong Yin, Tonghai Liu

**Affiliations:** ^1^ The Second People's Hospital of Liaocheng The Second Hospital of Liaocheng Affiliated to Shandong First Medical University Shandong China; ^2^ Department of Radiation Oncology Shandong Cancer Hospital and Institute Shandong First Medical University and Shandong Academy of Medical Sciences Shandong China

**Keywords:** 4D CT, breathing motion, deformable registration, dosimetry, hepatocellular carcinoma

## Abstract

**Purpose:**

To investigate the effect of breathing motion on dose distribution for hepatocellular carcinoma (HCC) patients using four‐dimensional (4D) CT and deformable registration.

**Methods:**

Fifty HCC patients who were going to receive radiotherapy were enrolled in this study. All patients had been treated with transarterial chemoembolization beforehand. Three‐dimensional (3D) and 4D CT scans in free breathing were acquired sequentially. Volumetric modulated arc therapy (VMAT) was planned on the 3D CT images and maximum intensity projection (MIP) images. Thus, the 3D dose (Dose‐_3D_) and MIP dose (Dose‐_MIP_) were obtained, respectively. Then, the Dose‐_3D_ and Dose‐_MIP_ were recalculated on 10 phases of 4D CT images, respectively, in which the end‐inhale and end‐exhale phase doses were defined as Dose‐_3D‐EI_, Dose‐_3D‐EE_, Dose‐_MIP‐EI_, and Dose‐_MIP‐EE_. The 4D dose (Dose‐_4D‐3D_ and Dose‐_4D‐MIP_) were obtained by deforming 10 phase doses to the end‐exhale CT to accumulate. The dosimetric difference in Dose‐_3D_, Dose‐_EI3D_, Dose‐_EE3D_, Dose‐_4D‐3D_, Dose‐_MIP_, Dose‐_EIMIP_, Dose‐_EEMIP_, and Dose‐_4D‐MIP_ were compared to evaluate the motion effect on dose delivery to the planning target volume (PTV) and normal liver.

**Results:**

Compared with Dose‐_3D_, PTV D99 in Dose‐_EI3D_, Dose‐_EE3D_ and Dose‐_4D‐3D_ decreased by an average of 6.02%, 1.32%, 2.43%, respectively (*P* < 0.05); while PTV D95 decreased by an average of 3.34%, 1.51%, 1.93%, respectively (*P* < 0.05). However, CI and HI of the PTV in Dose‐_3D_ was superior to the other three distributions (*P* < 0.05). There was no significant differences for the PTV between Dose‐_EI_ and Dose‐_EE_, and between the two extreme phase doses and Dose‐_4D_ (*P*> 0.05). Negligible difference was observed for normal liver in all dose distributions (*P*> 0.05).

**Conclusions:**

Four‐dimensional dose calculations potentially ensure target volume coverage when breathing motion may affect the dose distribution. Dose escalation can be considered to improve the local control of HCC on the basis of accurately predicting the probability of radiation‐induced liver disease.

## 1 Introduction

For patients with unresectable hepatocellular carcinoma (HCC), transarterial chemoembolization (TACE) followed by volumetric modulated arc therapy (VMAT) is a safe and effective treatment which can achieve better outcomes than either of these therapies alone.^1^ However, radiation‐induced liver disease (RILD) remains the limiting factor of dose escalation for the target volume. Abdominal organs can move and deform significantly during breathing. In order to estimate the RILD, it is important to evaluate the effect of breathing motion on dose distribution.

The majority of patients are often treated in free breathing, which may bring the geometric uncertainties to either the target volume or normal liver.^2^ Further, the interplay between the incident beam and tumor motion derived from breathing motion could potentially introduce dosimetric error, leading to difference between the dose distributions from 3D static plan and the radiation dose actually delivered.^3^ The 3D plan in free breathing could not reflect the dose to the target volume and normal liver during whole breathing cycle. This would make the prediction to the probability of RILD inaccurate.

Recently, four‐dimensional (4D) CT has revealed the real motion information of regions of interest (ROIs) during breathing.^4^ 4D CT has been used in precision radiotherapy of lung cancer, HCC and pancreatic carcinoma, which were affected by breathing motion significantly.^5^ It had been proved that the application of 4D CT in HCC radiotherapy could improve the target localization, and the potential clinical benefits have been summarized.^6^ To estimate real dose delivered to the patients, 4D accumulated dose using 4D CT and deformable registration (DR) has been studied to explicitly account for the effects of breathing motion on dose distribution.^7^

DR can model the tissue deformation by solving the voxel‐to‐voxel transformations between two images. ^8^ Brock *et al*. demonstrated the accuracy of target localization using a biomechanical model‐based multi‐organ registration (Morfeus) algorithm.^9^ This algorithm would also be able to accumulate dose over multiple fractions in the presence of breathing motion. A 4D dose distribution can be obtained by deforming the dose matrix from different phases of 4D CT onto the reference image on the basis of anatomic correspondence and accumulating the dose matrices. Compared with 3D dose, the 4D accumulated dose could involve the dose delivered to the patients during whole breathing cycle.

In this study, we investigated the feasibility of 4D dose accumulation for the target volume and normal liver in radiotherapy of HCC using Morfeus, and analyzed the dosimetric differences between 3D and 4D dose distributions. In addition, end‐inhale and end‐exhale phase doses were calculated to evaluate the dosimetric effects of rigid motion on the target and normal liver.

## 2 Materials and Methods

### 2.1 Patient selection

Fifty HCC patients were consecutively included in this study. All patients were treated on a Research Ethics Board of our institution approved protocol, with written informed consent obtained from them. The inclusion criteria included the patients with unsuitable or unwilling resection, Child‐Pugh liver score A, KPS more than 80. All patients had received TACE then re‐planned by VMAT, each with complete lipiodol retention. The patients included 39 men and 11 women, with a median age of 63.5 years (range, 41–77 years). Of the 50 tumors, 22 located in the left lobe of the liver, 28 in the right lobe.

### 2.2 Image acquisition

Patients were supine with arms above head and immobilized using an evacuated cushion. Three‐dimensional and 4D CT scans for all of the patients during free breathing were sequentially obtained using a multislice CT scanner (Philips Medical Systems, Highland Heights, OH, USA). Based on the breath signal obtained from the real‐time positioning management system (Varian Medical Systems, Palo Alto, CA, USA), 4D CT data were sorted into 10 breathing phases with equal duration (CT00–CT90). CT00 was defined as end‐inhalation and CT50 as end‐exhalation. Before the CT image acquisition, patients were coached to breath in a regular and reproducible manner. All CT images were transferred to the Raystation treatment planning system, version 3.99.0.7 (RaySearch Laboratories, Sweden) for delineation and dose calculation.

### 2.3 ROIs delineation and treatment planning

The GTVs and liver were delineated on all CT images. The GTV contours were determined by the radiation oncologists with consultations from the radiologists. The internal gross tumor volume (IGTV) was generated by merging 10 GTVs on all phases of 4D CT images. PTV in 3D images were obtained from GTV plus a 1.2‐cm margin in head‐foot direction and 1.0‐cm margin in other direction.^10^ PTV in MIP images were obtained from GTV plus a 0.8‐cm margin in each direction. Normal liver was defined to subtract PTV from the whole liver. The treatment planning goal was to achieve 98% of PTV coverage with 100% of the prescription dose, while 10% of the volume of PTV not exceeding 110% of the prescription dose. The mean dose of normal liver was limited to 28 Gy. The maximal dose of stomach and duodenum was limited to 45 Gy, with the volume receiving over 25 Gy less than 5 cm^3^. For the spinal cord, the maximal dose to a point was less than 45 Gy.^11^ For all patients, individualized VMAT plans were generated with four parts of the arc and 6‐MV photon energy at the Varian VitalBeam Linear Accelerator (Varian Medical Systems, Inc., Palo Alto, CA, USA). The prescription dose was 50 Gy in five fractions.

### 2.4 DR

Dose accumulation requires that tissues be accurately tracked between images. In this study, a biomechanical DR based on the Morfeus method was used.^12^ This algorithm deforms the structures by solving a linear elasticity problem using the finite element model. The problem is set up by controlling ROIs represented by meshes with vertex‐to‐vertex correspondence in the reference and target image sets. The meshes can be generated with model‐based segmentation or with dedicated tools from contours. For controlling ROIs representing interior structures, including liver, PTV, GTV, the interface between the structure and the surrounding tissue can be modeled as either fixed or sliding. Thus the biomechanical DR is completely geometry‐based and does not incorporate any grayscale information. The accuracy of this method for all deformed tissues is less than 0.2 cm.^12^

### 2.5 Dose calculations

For this study, dose was accumulated and compared in the Morfeus environment. The 3D dose (Dose‐_3D_) was calculated on the 3D CT image using collapsed cone convolution superposition algorithm in the TPS. The 3D dose (Dose‐_3D_) was recalculated on each phase of 4D CT images and single‐phase dose distribution, as well as dose‐volume histogram (DVH) was generated. The end‐inhale and end‐exhale phase doses, defined as Dose‐_EI_ and Dose‐_EE_, respectively, were selected for comparison to account for the effects of rigid motion on dose distribution. After doses were recomputed, the Morfeus algorithm was used to deform the other nine phase doses of 4D CT to the end‐exhale phase. Dose distributions at different breathing phases were accumulated on the end‐exhale phase and 4D dose (Dose‐_4D_) was obtained. For MIP images, the same operation was performed as the above steps.

### 2.6 Plan evaluation

For PTV, the D99, D95, and D1 were defined as the least doses received by 99%, 95%, and 1% of the target volume. The homogeneity index (HI) was described by using the ratio of D2 to D98, as follows:

$$\text{HI}=\frac{D2‐D98}{prescription\;dose}$$

The conformal index (CI) was calculated as follows:

$$\text{CI}=\frac{TV_{\mathrm{RI}}}{\mathrm{TV}}\times \frac{TV_{\mathrm{RI}}}{V_{\mathrm{RI}}}$$

where TV_RI_ is the target volume covered by the prescription dose, TV is the target volume, and V_RI_ is the volume of the prescription dose. The mean dose delivered to the normal liver (D_mean_), V5, V10, V20, V30, and V40 were also evaluated, where V_x_ represents the percentage of the volume of x Gy in the normal liver.

### 2.7 Statistical analysis

**Fig. 1:**
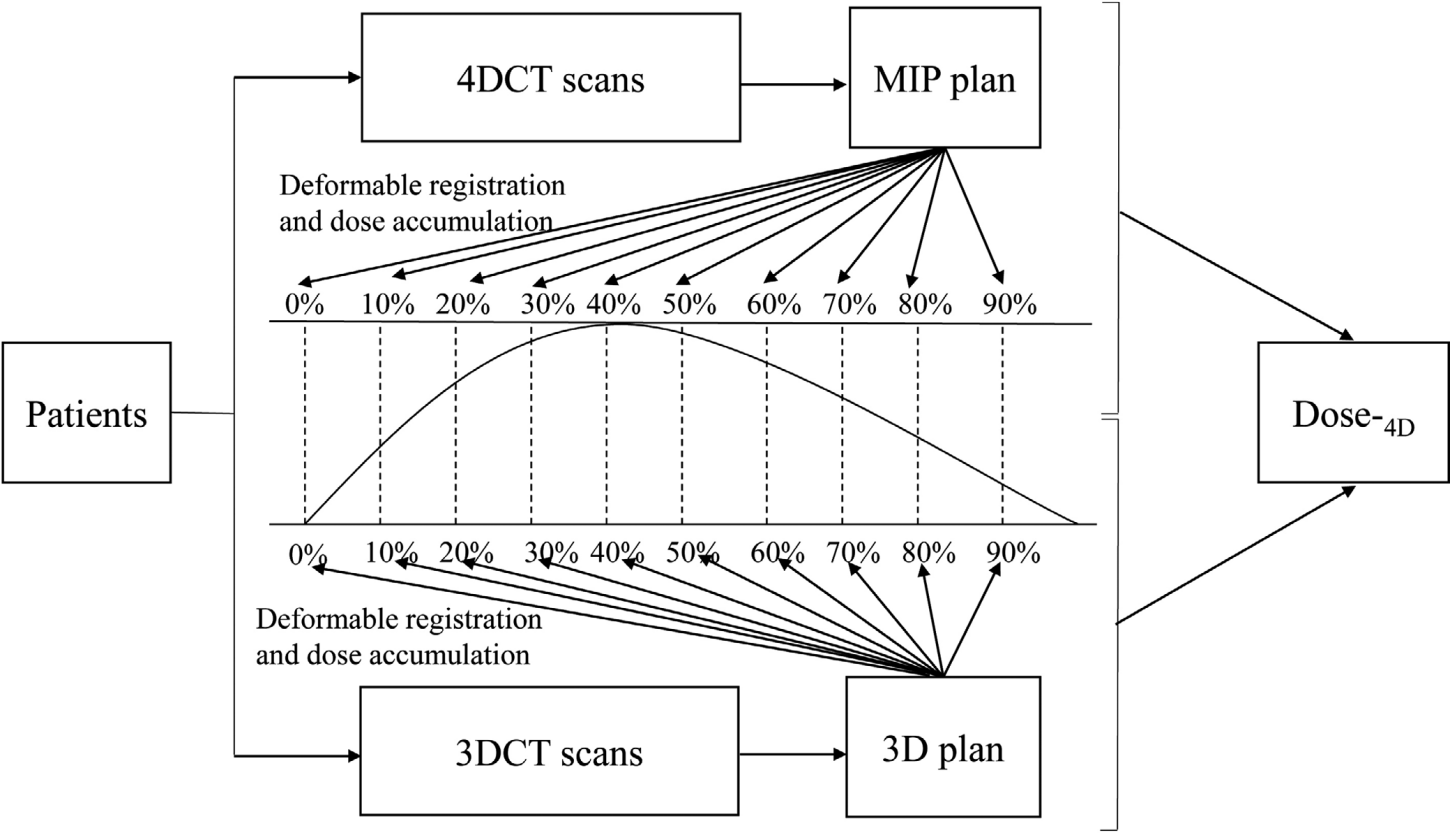
Flow chart of this study.

The data were analyzed using SPSS 17.0 software package (IBM, Chicago, IL, USA). The dosimetric parameters of Dose‐_3D_, Dose‐_EI_, Dose‐_EE_ and Dose‐_4D_ for PTV and normal liver were compared using Friedman test. The Wilcoxon test was used for the pairwise data. Differences were considered significant at *P* < 0.05. The flow chart of this study was depicted in Fig. 1.

## 3 Results

### 3.1 ROIs volumes and motion amplitude

The volumes of GTV and liver delineated on the 3D CT image were 58.35 ± 35.31 cm^3^ and 1575.24 ± 302.67 cm^3^, respectively; while the average volumes of GTV and liver from all phases of 4D CT images were 52.14 ± 48.26 cm^3^ and 1501.53 ± 294.27 cm^3^, respectively. No significant differences were found between either GTVs or livers from the 3D and 4D images (*P*> 0.05). Motion amplitude was determined as the distance between the ROI centroids on end‐inhale and end‐exhale phase images. The motion amplitude of GTV and liver was 0.66 ± 0.47 cm and 0.49 ± 0.58 cm, respectively.

### 3.2 Registration evaluation

**Fig. 2:**
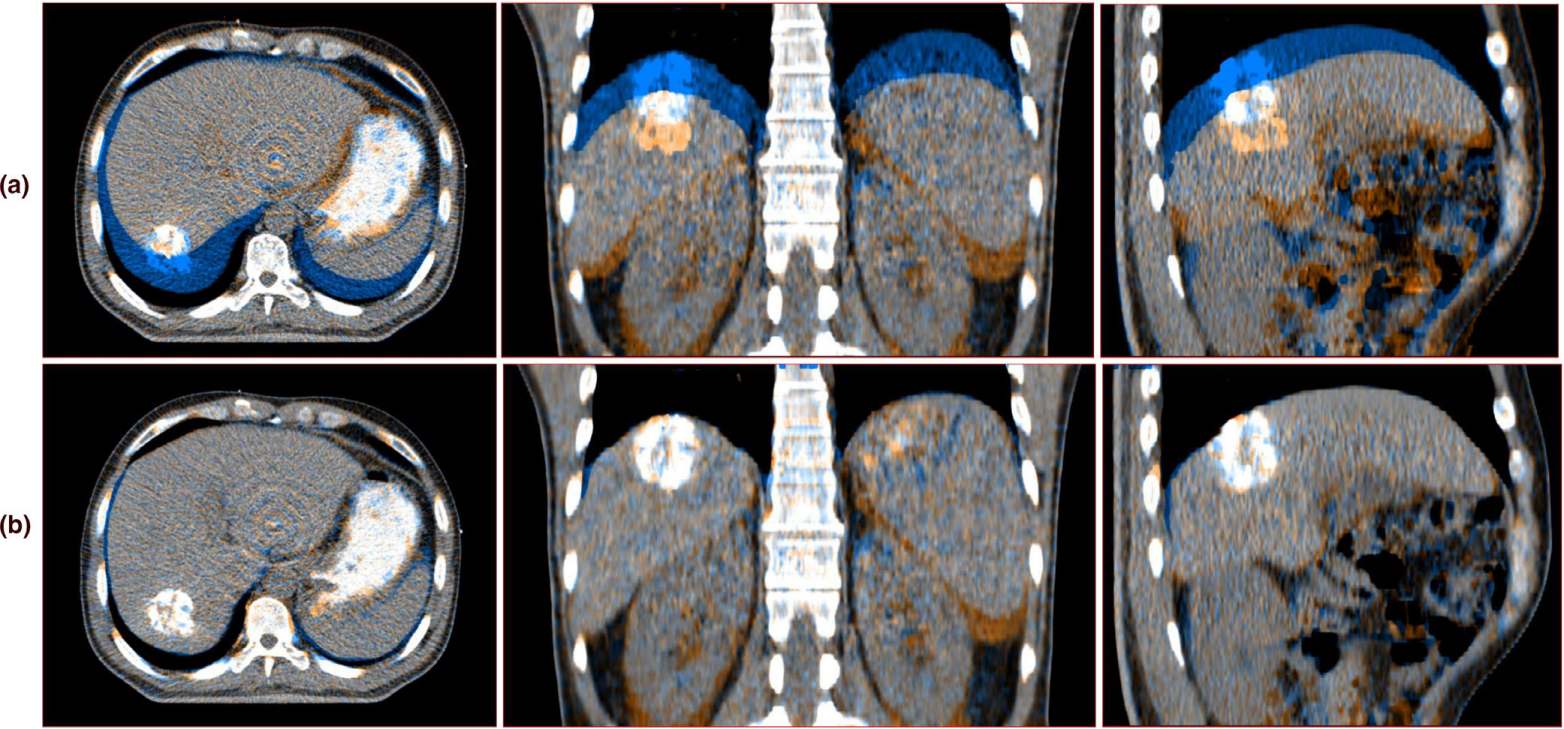
The fusion views of EI and EE phase images. (a) Before DR (b) after DR. The blue represents the EE phase image, and the orange represents the EI phase image (left to right: transversal, coronal, and sagittal).

**Fig. 3:**
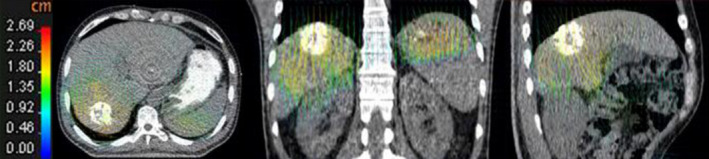
Deformable vector fields from EI phase image to EE phase image. Displacement field color table shows the magnitude, in which the blue indicates the least magnitude and the red the most magnitude (left to right: transversal, coronal, and sagittal).

A quantitative assessment have been completed previously by Brock *et al*.^12^ Further, visual inspection was performed to test the registration differences between target and reference images. Figure 2 displays the results of GTV showing the largest breathing amplitude. Inspection of these images before and after registration showed the good performance of the algorithm, and differences were substantially reduced. Note the good agreement of the liver and tumor after registration. The corresponding deformable vector fields were shown in Fig. 3. The color and arrows corresponds to the magnitude and direction of the vector in each point.

### 3.3 Dosimetric indices for the PTV

**Fig. 4:**
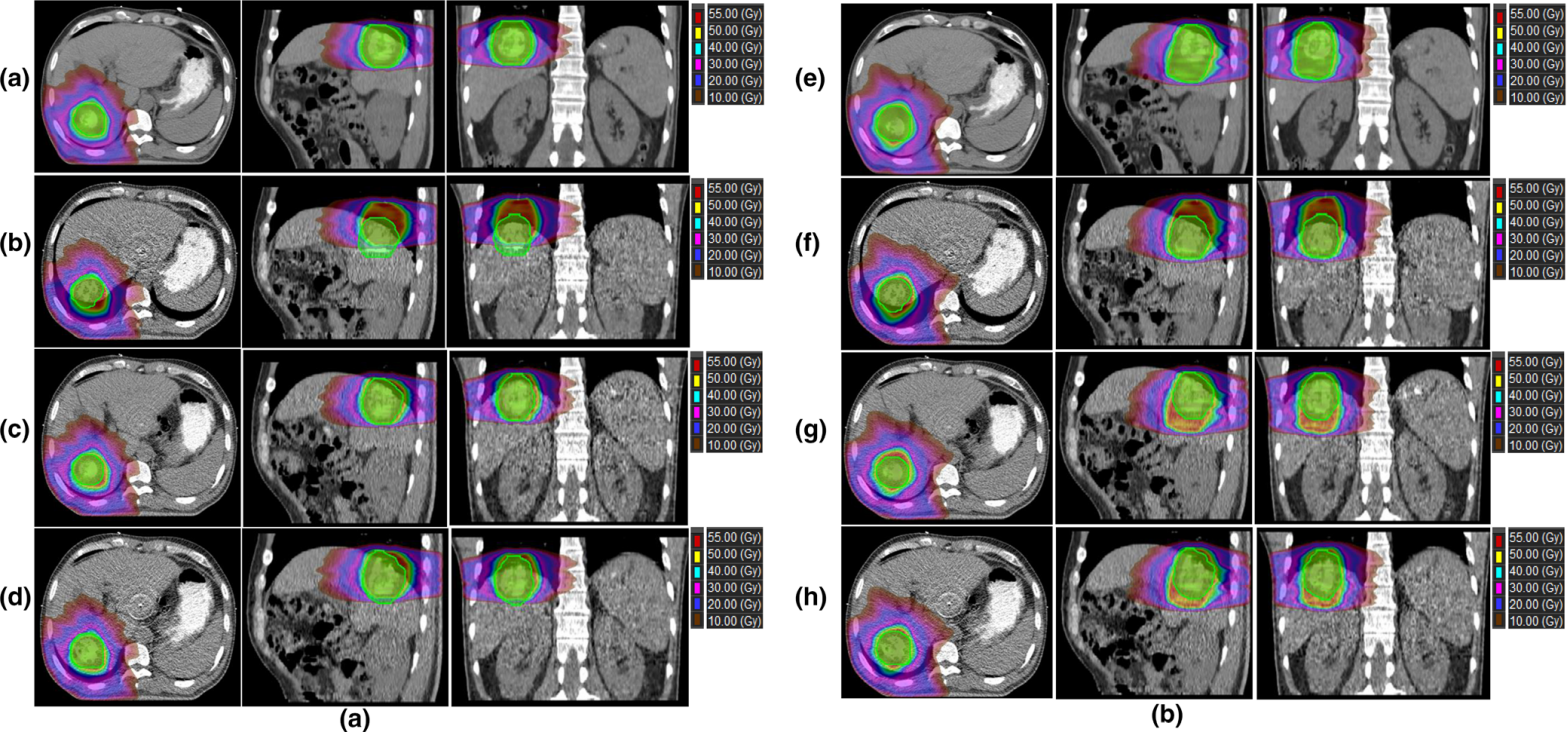
A is the PTV coverage in Dose‐_3D_, Dose‐_3D‐EI_, Dose‐_3D‐EE_,and Dose‐_3D‐4D._ B is the PTV coverage in Dose‐_MIP_, Dose‐_MIP‐EI_, Dose‐_MIP‐EE_, and Dose‐_MIP‐4D._

**Fig. 5:**
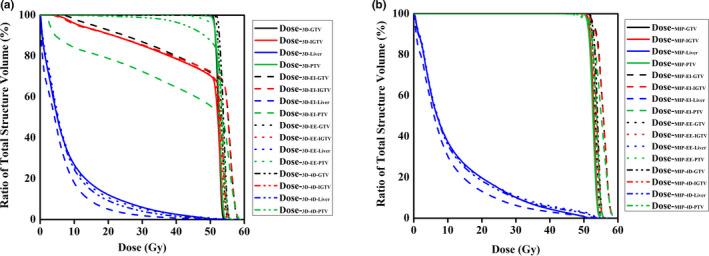
A comparison of DVHs in different dose distributions.

As shown in Table 1, for PTV D99 and D95, Dose‐_3D_ was higher than those from Dose‐_EI‐3D_, Dose‐_EE‐3D_, and Dose‐_4D‐3D_; while for PTV CI and HI, Dose‐_3D_ had the optimal values, compared with the other dose distributions (*P* < 0.05). No significant differences were observed for PTV D99, D95, CI, and HI among Dose‐_4D_, Dose‐_EI_, and Dose‐_EE_ (*P*> 0.05). Figure 4(a) displays the decrease of the PTV coverage in Dose‐_3D‐EI_, Dose‐_3D‐EE_, and Dose‐_3D‐4D_ due to breathing motion compared with Dose‐_3D_. Underdosing of these PTVs was also displays as the wider shoulder in the DVHs in Fig. 5(a).

**Table 1:** Dosimetric indices for PTV in Dose‐_3D_, Dose‐_3D‐EI_, Dose‐_3D‐EE_, and Dose‐_3D‐4D._

Indices	Dose‐_3D_	Dose‐_3D‐EI_	Dose‐_3D‐EE_	Dose‐_3D‐4D_	*χ^2^*	*P*
D99 (Gy)	49.65 ± 0.20	43.63 ± 4.47	48.33 ± 8.18	47.22 ± 4.71	28.62	0.00
D95 (Gy)	50.67 ± 0.25	47.43 ± 3.09	49.16 ± 4.13	48.74 ± 4.01	29.37	0.00
D1 (Gy)	53.95 ± 0.22	57.19 ± 1.21	54.26 ± 1.12	54.46 ± 1.05	6.28	0.14
CI	0.85 ± 0.16	0.54 ± 0.37	0.62 ± 0.28	0.68 ± 0.27	34.26	0.00
HI	0.07 ± 0.02	0.21 ± 0.15	0.19 ± 0.21	0.16 ± 0.12	32.15	0.00

As shown in Table 2, for PTV D99 and D95, Dose‐_MIP_ was almost equally to Dose‐_EI‐MIP_, Dose‐_EE‐MIP_, and Dose‐_4D‐MIP_; while for PTV CI and HI, Dose‐_MIP_ had the superior values, compared with the other dose distributions (*P* < 0.05). No significant differences were observed for PTV D99, D95, CI, and HI among Dose‐_4D‐MIP_, Dose‐_EI‐MIP_, and Dose‐_EE‐MIP_ (*P*> 0.05). Figure 4(b) displays the PTV coverage in Dose‐_MIP_, Dose‐_MIP‐EE_, Dose‐_MIP‐EE_, and Dose‐_MIP‐4D_. Figure 5(b) shows the DVHs of different structures in all plans.

**Table 2:** Dosimetric indices for PTV in Dose‐_MIP_, Dose‐_MIP‐EI_, Dose‐_MIP‐EE_, and Dose‐_MIP‐4D._

Indices	Dose‐_MIP_	Dose‐_MIP‐EI_	Dose‐_MIP‐EE_	Dose‐_MIP‐4D_	*χ^2^*	*P*
D99 (Gy)	49.48 ± 0.22	49.23 ± 1.05	49.13 ± 1.05	49.22 ± 3.61	5.13	0.39
D95 (Gy)	50.46 ± 0.21	51.23 ± 4.18	50.37 ± 2.81	50.83 ± 3.22	4.31	0.47
D1 (Gy)	53.87 ± 0.19	54.61 ± 2.39	54.06 ± 1.09	54.01 ± 0.85	5.14	0.55
CI	0.84 ± 0.14	0.72 ± 0.58	0.75 ± 0.34	0.78 ± 0.17	6.93	0.61
HI	0.07 ± 0.03	0.11 ± 0.16	0.11 ± 0.31	0.11 ± 0.13	7.44	0.53

### 3.4 Dosimetric indices for normal liver

Table 3 shows the mean differences for D_mean_, V5, V10, V20, V30, and V40 in Dose‐_3D_, Dose‐_EI‐3D_, Dose‐_EE‐3D_, and Dose‐_4D‐3D_. There was no significant difference between Dose‐_3D_, Dose‐_EE‐3D_, and Dose‐_4D‐3D_ (*P*> 0.05). In Dose‐_EI‐3D_, a decrease of normal liver mean dose is statistical difference (P < 0.05).

**Table 3:** Dosimetric indices for normal liver in Dose‐_3D_, Dose‐_3D‐EI_, Dose‐_3D‐EE_, and Dose‐_3D‐4D._

Indices	Dose‐_3D_	Dose‐_3D‐EI_	Dose‐_3D‐EE_	Dose‐_3D‐4D_	*χ^2^*	*P*
D_mean_ (Gy)	13.31 ± 4.26	11.43 ± 6.25	12.52 ± 6.35	12.18 ± 6.38	2.55	0.48
V5 (%)	63.26 ± 9.64	59.43 ± 14.26	62.28 ± 13.54	63.04 ± 13.24	3.24	0.35
V10 (%)	45.39 ± 10.22	38.47 ± 12.15	44.38 ± 13.63	43.41 ± 14.26	1.07	0.63
V20 (%)	19.17 ± 12.31	14.78 ± 13.19	18.21 ± 13.55	17.38 ± 13.26	2.54	0.57
V30 (%)	8.16 ± 6.23	6.17 ± 5.96	8.02 ± 6.83	7.92 ± 6.03	3.23	0.46
V40 (%)	6.17 ± 3.31	5.36 ± 4.85	5.94 ± 4.83	5.81 ± 4.85	2.86	0.41

Table 4 shows the mean differences for D_mean_, V5, V10, V20, V30, and V40 in Dose‐_MIP_, Dose‐_EI‐MIP_, Dose‐_EE‐MIP_, and Dose‐_4D‐MIP_, with none of these differences being significant (*P*> 0.05). In Fig. 5(b), the four dose distributions had very similar DVHs, with a relative small spread.

**Table 4:** Dosimetric indices for normal liver in Dose‐_MIP_, Dose‐_MIP‐EI_, Dose‐_MIP‐EE_, and Dose‐_MIP‐4D._

Indices	Dose‐_MIP_	Dose‐_MIP‐EI_	Dose‐_MIP‐EE_	Dose‐_MIP‐4D_	*χ^2^*	*P*
D_mean_ (Gy)	14.25 ± 4.19	14.11 ± 4.56	14.22 ± 4.15	14.18 ± 4.26	1.22	0.65
V5 (%)	65.13 ± 13.72	63.24 ± 13.28	65.23 ± 13.61	64.49 ± 13.15	5.24	0.43
V10 (%)	41.39 ± 13.55	38.26 ± 13.19	40.86 ± 12.98	40.63 ± 13.12	1.22	0.54
V20 (%)	33.17 ± 12.86	30.72 ± 11.35	32.89 ± 12.13	32.45 ± 12.11	2.86	0.47
V30 (%)	15.28 ± 8.24	13.22 ± 8.68	15.01 ± 8.35	14.95 ± 8.22	3.29	0.48
V40 (%)	8.16 ± 6.25	7.26 ± 6.34	7.87 ± 6.37	7.95 ± 6.12	4.35	0.43

## 4 Discussion

With the development of 4D CT and the mature of DR, the 4D dose accumulation has become feasible at planning stage. This study indicated the 4D dose distribution have a different effect on the target and normal liver in free breathing, compared with the 3D static plan in HCC patients. The dose delivered to the target fall short of the prescription dose, therefore, a 5.64% dose escalation could be expected to improve local control, while the doses delivered to the normal liver were negligible. The approach of 4D dose accumulation yields a more actual and accurate prediction of dose delivered.^13^, ^14^ This approach is especially beneficial for the clinic to effectively predict the probability of RILD.

In 3D plans, differences between Dose‐_3D_ and Dose‐_4D_ derived largely from geometric uncertainties induced by breathing motion. Adding margins to form PTV may incorporate the setup uncertainties, but this expansion cannot account for breathing motion. Therefore, breathing dose should be accumulated to accurately estimate the dose‐volume relationships during radiotherapy of HCC.

For the target volume, departure from the relatively static geometry to moving anatomy resulted in different dose to the PTV. Especially at the periphery of the PTV, the dose gradients are more sensitive to geometric errors from breathing. Lujan *et al*. reported that small breathing motion (<3mm) led to small changes in the dose distributions, but patient with over 1.0 cm of motion had significant changes to most tissues.^15^ It can be expected that patients with significant breathing motion may benefit more from DR, potentially reducing dose‐toxicity risks and improving dose escalation. For normal liver, motion‐induced variations were relatively small and could be negligible. As a parallel organ of large volume, any severe perturbations of the dose likely occur in small regions and thus the effect of these perturbations on the total organ dose response may be too small to produce clinically significant changes.

The results did not support large differences between Dose‐_EI_ and Dose‐_EE_, or between the two extreme phase doses and the Dose‐_4D_. Considering the average volume of 58.35cm^3^ and the excursion of 0.66cm, the volumes and the motion amplitude of GTV may be small. Therefore, if more cases with larger breathing motion is included to experiment further, the significant changes may be observed among Dose‐_EI_, Dose‐_EE_, and the Dose‐_4D_. Velec *et al*. demonstrated the changes from −5.1% to 8.3% in GTV and from −0.2% to 1.0% in normal liver between rigid and deformable dose accumulation in stereotactic liver cancer radiotherapy.^16^ In our study, a 3.02% and 1.32% decrease for PTV was observed in Dose‐_EI‐3D_ and Dose‐_EE‐3D_, respectively, compared with the prescription dose. It may not be accurate to account for the actual dose distribution with the single‐phase dose of 4D CT.

The motion effect on dose distribution with DR of 4D CT has been studied less for liver radiotherapy. Velec *et al*. accumulated 4D dose by deforming exhale CT to inhale CT to model intermediate breathing motion. Relative to 3D dose, minimal dose to GTV fluctuated between −14% and 8%, and mean dose to normal liver between −3% and 4%.^16^ However, the intermediate phase motion modeling of the 4D CT data may not reflect the actual breathing motion, and the final dose distribution could be unreliable. Flampouri *et al*. reported that three phase accumulated dose had a 3% difference from 10 phase accumulated dose.^17^ Rosu *et al*. described the difference between 10 phase and two phase dose were about 2%.^18^ Jung *et al*. summed 10 phase doses of 4D CT data in liver cancer patients. The mean dose and generalized equivalent uniform dose for normal liver increased by 3.1% ± 3.3% and 2.8% ± 3.3% respectively, with none of difference in GTV coverage showing significance.^19^ In their study, the end‐exhale phase image was defined as reference image for treatment planning and dose accumulation. In clinical practice, many patients received radiotherapy during free breathing. A 3D CT image in free breathing will often be used for treatment planning instead of any phase of 4D CT images.

In contrast, our approach is consistent with the clinical practice. Furthermore, the TACE with iodized oil made the liver tumor visualized clearly during the process of DR. Thus, the lipiodol retention area can be the important landmark to evaluate the accuracy of the DR algorithm. Figure 2 shows the lipiodol retention area overlapped better after DR, suggesting the registration error would be acceptable for the mapping of ROIs contours and dose. Brock quantitatively evaluated that the average accuracy for various DR algorithms using the same 4D CT datasets, was to be less than 0.25 cm.^20^

Several limitations of this study should be mentioned. First, 4D dose was accumulated on the basis of equal weighting of each 4D CT phase. It may be more accurate to develop a time‐weighted sorting scheme to describe the breathing cycle and to predict the accumulated dose in free breathing. However, Rosu *et al*. showed that the varied accumulated dose distributions were dose together, with no clear separation, irrespective of the accumulation method.^18^ Another limitation was that we did not evaluate the biological effect of the 4D dose. It was reported that significant increases (decreases) in liver NTCP occurred for tumors located toward the bottom (top) of the liver when patients scanned at exhalation.^21^ As described previously, the NTCP of normal liver in this study was less than 5% when we set a criteria for ROIs.^11^

This temporal variation in anatomy highlights the importance of breathing motion management techniques. Active breathing control or gating, if suitable, is an effective method to reduce the magnitude of tumor motion.^22^, ^23^ Through the use of these techniques for better control of breathing motion, the dose differences will be smaller. However, the reproducibility of these techniques should not be neglected.

## 5 Conclusion

Four‐dimensional dose accumulation can be implemented with the availability of 4D CT and deformable registration in the presence of breathing motion. Compared with 3D static plan, the accumulated dose can reflect the more real dose to the target and the normal liver. And it is important for HCC patients to accurately predict the probability of RILD and facilitate the further safe dose escalation.

## Conflicts of interest

The authors have no other relevant conflicts of interest to disclose.

## Author Contribution

Conceived and designed the experiments: TH Liu, H Xu. Performed the experiments: TH Liu, H Xu. Analyzed the data: H Xu, GZ Gong, Y Yin. Wrote the paper: TH Liu, H Xu.
